# Executive Function Deficits in Genetic Frontotemporal Dementia: Results From the GENFI Study

**DOI:** 10.1212/NXG.0000000000200248

**Published:** 2025-07-21

**Authors:** Lucy Louise Russell, Arabella Bouzigues, Rhian S. Convery, Phoebe H. Foster, Eve Ferry-Bolder, David M. Cash, John C. Van Swieten, Lize Corrine Jiskoot, Harro Seelaar, Fermin Moreno, Raquel Sánchez-Valle, Robert Laforce, Caroline Graff, Mario Masellis, Maria Carmela Tartaglia, James B. Rowe, Barbara Borroni, Elizabeth Finger, Matthis Synofzik, Daniela Galimberti, Rik Vandenberghe, Alexandre de Mendonça, Christopher Butler, Alexander Gerhard, Simon Ducharme, Isabelle Le Ber, Isabel Santana, Florence Pasquier, Johannes Levin, Sandro Sorbi, Markus Otto, Jonathan Daniel Rohrer, for the Genetic FTD Initiative (GENFI), Aitana Sogorb Esteve

**Affiliations:** 1 Department of Neurodegenerative Disease, Dementia Research Centre, UCL Institute of Neurology, London, United Kingdom;; 2 Centre for Medical Image Computing, University College London, United Kingdom;; 3 Department of Neurology, Erasmus Medical Centre, Rotterdam, Netherlands;; 4 Cognitive Disorders Unit, Department of Neurology, Donostia University Hospital, San Sebastian, Gipuzkoa, Spain;; 5 Neuroscience Area, Biodonostia Health Research Institute, San Sebastian, Gipuzkoa, Spain;; 6 Alzheimer's disease and Other Cognitive Disorders Unit, Neurology Service, Hospital Clínic de Barcelona, Institut d'Investigacións Biomèdiques August Pi I Sunyer, University of Barcelona, Spain;; 7 Département des Sciences Neurologiques, CHU de Québec, and Faculté de Médecine, Clinique Interdisciplinaire de Mémoire, Université Laval, Canada;; 8 Center for Alzheimer Research, Division of Neurogeriatrics, Department of Neurobiology, Care Sciences and Society, Bioclinicum, Karolinska Institutet, Solna, Sweden;; 9 Unit for Hereditary Dementias, Theme Aging, Karolinska University Hospital, Solna, Sweden;; 10 Sunnybrook Health Sciences Centre, Sunnybrook Research Institute, University of Toronto, Canada;; 11 Tanz Centre for Research in Neurodegenerative Diseases, University of Toronto, Canada;; 12 Department of Clinical Neurosciences and Cambridge University Hospitals NHS Trust and Medical Research Council Cognition and Brain Sciences Unit, University of Cambridge, United Kingdom;; 13 Centre for Neurodegenerative Disorders, Department of Clinical and Experimental Sciences, University of Brescia, Italy;; 14 Department of Clinical Neurological Sciences, University of Western Ontario, London, Canada;; 15 Department of Neurodegenerative Diseases, Hertie-Institute for Clinical Brain Research and Center of Neurology, University of Tübingen, Germany;; 16 Center for Neurodegenerative Diseases (DZNE), Tübingen, Germany;; 17 Department of Biomedical, Surgical and Dental Sciences, University of Milan, Italy;; 18 Fondazione IRCCS Ca' Granda, Ospedale Maggiore Policlinico, Milan, Italy;; 19 Laboratory for Cognitive Neurology, Department of Neurosciences, KU Leuven, Belgium;; 20 Neurology Service, University Hospitals Leuven, Belgium;; 21 Leuven Brain Institute, KU Leuven, Belgium;; 22 Faculty of Medicine, University of Lisbon, Portugal;; 23 Nuffield Department of Clinical Neurosciences, Medical Sciences Division, University of Oxford, United Kingdom;; 24 Division of Psychology Communication and Human Neuroscience Wolfson Molecular Imaging Centre, University of Manchester, United Kingdom;; 25 Department of Nuclear Medicine, Center for Translational Neuro- and Behavioral Sciences, University Medicine Essen, Germany;; 26 Department of Geriatric Medicine, Klinikum Hochsauerland, Arnsberg, Germany;; 27 Department of Psychiatry, Douglas Mental Health University Institute, McGill University, Montreal, Canada;; 28 Department of Neurology and Neurosurgery, McConnell Brain Imaging Centre, Montreal Neurological Institute, McGill University, Montreal, Canada;; 29 Paris Brain Institute—Institut du Cerveau—ICM, Inserm U1127, CNRS UMR 7225, AP-HP–Hôpital Pitié-Salpêtrière (DMU Neurosciences Paris 6), Sorbonne Université, France;; 30 Département de Neurologie, Centre de référence des démences rares ou précoces, IM2A, AP-HP–Hôpital Pitié-Salpêtrière (DMU Neurosciences Paris 6), Paris, France;; 31 Département de Neurologie, AP-HP–Hôpital Pitié-Salpêtrière (DMU Neurosciences Paris 6), France;; 32 Neurology Department, Centro Hospitalar e Universitário de Coimbra, Portugal;; 33 Faculty of Medicine, University of Coimbra, Portugal;; 34 Center for Innovative Biomedicine and Biotechnology (CIBB), University of Coimbra, Portugal;; 35 Univ Lille, France;; 36 Inserm 1172, Lille, France;; 37 CHU Lille, CNR-MAJ, Labex Distalz, LiCEND, France;; 38 Neurologische Klinik und Poliklinik, Ludwig-Maximilians-Universität, Munich, Germany;; 39 German Center for Neurodegenerative Diseases (DZNE), Munich, Germany;; 40 Munich Cluster of Systems Neurology, Germany;; 41 Department of Neurofarba, University of Florence, Italy;; 42 IRCCS Fondazione Don Carlo Gnocchi, Florence, Italy; and; 43 Department of Neurology, University of Ulm, German.

## Abstract

**Background and Objectives:**

Executive dysfunction is a core feature of frontotemporal dementia (FTD). While there has been extensive research into such impairments in sporadic FTD, there has been little research in the familial forms.

**Methods:**

Seven hundred fifty-two individuals were recruited in total: 214 *C9orf72*; 205 *progranulin (GRN)* and 86 *microtubule associated protein tau (MAPT)* mutation carriers, stratified into asymptomatic, prodromal, and fully symptomatic; and 247 mutation-negative controls. Attention and executive function were measured using the Weschler Memory Scale-Revised (WMS-R) Digit Span Backwards (DSB), Wechsler Adult Intelligence Scale-Revised Digit Symbol task, Trail Making Test Parts A and B, and the Delis-Kaplan Executive Function System Color Word Interference Test. Linear regression models with bootstrapping were used to assess differences between groups. Correlation of task score with disease severity was also performed, as well as an analysis of the neuroanatomical correlates of each task.

**Results:**

Fully symptomatic *C9orf72*, *GRN*, and *MAPT* mutation carriers were significantly impaired on all tasks compared with controls (all *p* < 0.001), except on the WMS-R DSB in the *MAPT* mutation carriers (*p* = 0.147). While asymptomatic and prodromal *C9orf72* individuals also demonstrated differences compared with controls, neither the *GRN* or *MAPT* asymptomatic or prodromal mutation carriers showed significant deficits. All tasks were significantly correlated with disease severity in each of the genetic groups (all *p* < 0.001).

**Discussion:**

Some individuals with *C9orf72* mutations show difficulties with executive function from very early on in the disease and this continues to deteriorate with disease severity. By contrast, similar difficulties occur only in the later stages of the disease in *GRN* and *MAPT* mutation carriers. This differential performance across the genetic groups will be important in neuropsychological task selection in upcoming clinical trials.

## Introduction

Frontotemporal dementia (FTD) is a neurodegenerative disease that causes impairments in behavior and cognition. While a number of different changes in personality can occur, such as apathy, loss of empathy, and obsessive-compulsive behaviors,^1,2^ the core cognitive deficit is a change in executive function, a set of processes that includes inhibitory control, working memory, and cognitive flexibility.^3^

Executive function has been extensively studied in sporadic FTD, where it has been demonstrated that such abilities are commonly compromised. However, there have been fewer studies examining changes in the familial forms of FTD due to pathogenic variants in the progranulin (*GRN*) and microtubule-associated protein tau (*MAPT*) genes and a pathogenic expansion in chromosome 9 open reading frame 72 (*C9orf72*), which account for about one-third of all FTDs.^4-9^

The aim of this study was therefore to investigate executive function in a large cohort of presymptomatic and symptomatic individuals with familial FTD using participants from the Genetic FTD Initiative (GENFI). In particular, we explore whether there are differences across the 3 main genetic causes and whether deficits occur in the presymptomatic stages of the disease. Such information will be important in guiding task selection for inclusion and outcome measures in upcoming therapeutic trials.

## Methods

### Cohort

The fifth GENFI data freeze included 831 individuals from 25 sites in Europe and Canada. 752 of these individuals had completed at least 1 of the executive function tasks in the GENFI neuropsychological battery: 214 carried the *C9orf72* expansion, 205 had a *GRN* pathogenic variant, and 86 had a *MAPT* pathogenic variant (“mutation carriers” from here onward); 247 individuals were mutation-negative family members who acted as controls. The specific pathogenic gene variants are listed in eTable 1.

### Standard Protocol Approvals, Registrations, and Patient Consents

Ethical approval was gained at each of the individual sites, and all participants provided fully informed consent.

### Protocol

All participants underwent the standard GENFI protocol including the GENFI neuropsychological battery,^10,11^ as well as the Mini-Mental State Examination and the Clinical Dementia Rating (CDR) Dementia Staging Instrument with the National Alzheimer's Coordinating Center Frontotemporal Lobar Degeneration component (NACC FTLD). The latter generates 2 types of scores: a sum-of-boxes (SB) score and a global score that allows staging of the disease into 0—asymptomatic, 0.5—prodromal, and 1 or more (1+)—symptomatic (1 mild, 2 moderate, and 3 severe). Symptomatic individuals were diagnosed according to current criteria^12-14^: 91 had bvFTD (*C9orf72* = 49, *GRN* = 24, *MAPT* = 18), 20 had primary progressive aphasia (*C9orf72* = 3, *GRN* = 16, *MAPT* = 1), and 9 had FTD with amyotrophic lateral sclerosis (*C9orf72* = 9), while the other symptomatic participants consisted of smaller diagnostic groups including those with atypical parkinsonism. Demographic information is provided in Table 1.

**Table 1:** Demographic Information and Task Performance for Participants Split by Genetic Group and CDR Plus NACC FTLD Global Score

Genetic Group	Controls	C9orf72			GRN			MAPT		
CDR with NACC FTLD global	0	0	0.5	1+	0	0.5	1+	0	0.5	1+
N	247	110	36	68	129	31	45	48	14	24
% Male	43	42	39	65	35	48	51	40	29	67
Age at visit	45.3 (12.9)	44.2 (11.7)	49.3 (11.4)	62.2 (8.8)	45.9 (12.2)	51.8 (13.2)	63.5 (7.9)	39.3 (10.5)	45.7 (12.6)	57.3 (10.2)
Education	14.4 (3.3)	14.3 (3.0)	14.1 (2.5)	13.2 (3.7)	14.7 (3.4)	14.0 (4.0)	11.8 (3.3)	14.4 (3.4)	13.5 (2.4)	13.7 (3.9)
MMSE	29.3 (1.1)	29.2 (1.2)	28.6 (2.0)	23.7 (6.1)	29.5 (0.9)	28.5 (2.4)	21.0 (6.8)	29.5 (0.8)	28.2 (2.3)	23.7 (6.7)
CDR with NACC FTLD–SB	0.0 (0.0)	0.0 (0.0)	1.2 (0.8)	10.7 (5.5)	0.0 (0.0)	1.0 (0.8)	9.0 (5.5)	0.0 (0.0)	1.1 (0.8)	9.3 (5.5)
DSST (s)	58.5 (13.9)	**53.9 (12.6)**	52.5 (15.8)	**25.8 (13.2)**	58.4 (11.9)	50.8 (18.1)	**25.6 (14.7)**	61.7 (12.3)	56.7 (14.8)	**36.0 (15.4)**
DSB (n)	6.7 (2.2)	6.7 (2.2)	6.9 (2.6)	**3.6 (2.2)**	6.6 (2.1)	6.1 (2.5)	**3.2 (2.5)**	7.0 (2.1)	6.0 (2.0)	5.9 (2.8)
D-KEFS: Color (s)	28.6 (5.9)	**31.5 (8.3)**	**33.3 (10.3)**	**60.9 (23.8)**	29.1 (6.3)	31.7 (8.1)	**55.3 (26.9)**	28.0 (7.7)	31.5 (8.6)	**48.8 (19.1)**
D-KEFS: Word (s)	22.7 (5.6)	24.3 (7.9)	25.2 (7.2)	**38.8 (19.4)**	22.0 (5.2)	22.3 (6.5)	**35.2 (17.0)**	22.5 (6.9)	22.3 (5.2)	**31.8 (10.7)**
D-KEFS: Ink (s)	49.5 (12.1)	**59.4 (25.1)**	**61.6 (17.7)**	**129.0 (60.8)**	50.7 (16.2)	67.8 (48.2)	**123.1 (79.3)**	48.3 (20.2)	50.4 (13.8)	**89.1 (38.0)**
TMT A (s)	27.0 (12.3)	**30.2 (12.6)**	33.0 (18.2)	**67.2 (37.8)**	28.0 (9.7)	33.1 (24.1)	**80.2 (44.3)**	24.0 (9.4)	25.8 (8.5)	**52.9 (27.8)**
TMT B (s)	62.5 (31.4)	**75.4 (48.1)**	**91.9 (68.2)**	**196.2 (88.8)**	61.9 (24.0)	89.4 (75.5)	**224.2 (91.6)**	55.9 (22.5)	69.2 (36.3)	**167.2 (96.5)**

Means and SDs (in parentheses) are given. Results in bold show significant differences between the mutation carrier groups and controls.

CDR with NACC FTLD–SB: CDR. Dementia Staging Instrument with the National Alzheimer’s Coordinating Center Frontotemporal Lobar Degeneration component Sum-of-Boxes score; DSST: Wechsler Adult Intelligence Scale-Revised Digit Symbol substitution task; DSB: Weschler Memory Scale-Revised Digit Span Backwards; D-KEFS: Delis Kaplan Executive Function System (Color-Word Interference Test—Color = Color naming, Word = Word naming, Ink = Ink color naming); TMT: Trail Making Test (A = Part A; B = Part B).

Compared with controls, all 3 symptomatic groups were older (all *p* > 0.001) and so too was the *GRN* prodromal group (*p* = 0.004). The *MAPT* asymptomatic group was significantly younger than the controls (*p* = 0.001). All 3 symptomatic groups were also significantly older than their asymptomatic (all *p* < 0.001) and prodromal (all *p* < 0.003) counterparts. The *C9orf72* and *GRN* prodromal groups were also significantly older than their asymptomatic counterparts (*p* = 0.025 and *p* = 0.012, respectively). The *C9orf72* asymptomatic group was significantly older than the *MAPT* asymptomatic group (*p* = 0.016), and the *GRN* symptomatic group was significantly older than the *MAPT* symptomatic group (*p* = 0.040).

Differences in sex were found within the groups. The *C9orf72* and *MAPT* symptomatic groups consisted of significantly more men than the control group (*X*^2^ (1) = 9.8, *p* = 0.002; *X*^2^ (1) = 4.8, *p* = 0.028, respectively), and there were more men in the *C9orf72* symptomatic group than the other *C9orf72* groups (asymptomatic: *X*^2^ (1) = 8.8, *p* = 0.003; prodromal: *X*^2^ (1) = 6.4, *p* = 0.012). This was also the case for the *MAPT* symptomatic carriers (asymptomatic: X^2^ (1) = 4.7, *p* = 0.030; prodromal: *X*^2^ (1) = 5.1, *p* = 0.023).

When investigating education across the groups, the *GRN* symptomatic individuals had significantly lower levels of education than the control group (*p* < 0.001), *GRN* asymptomatic group (*p* < 0.01), and *C9orf72* (*p* = 0.035) and *MAPT* (*p* = 0.030) symptomatic groups. The *C9orf72* symptomatic group had a lower level of education than the control group (*p* = 0.010) and *C9orf72* asymptomatic group (*p* = 0.027).

### Executive Function Tasks

The following executive function tasks were included within the GENFI neuropsychology battery: the Wechsler Adult Intelligence Scale-Revised Digit Symbol substitution task (DSST),^15^ Weschler Memory Scale-Revised (WMS-R) Digit Span Backwards (DSB),^16^ Delis-Kaplan Executive Function System Color-Word Interference Test,^17^ and Trail Making Test (TMT) Parts A and B (TMT A and TMT B).

### Magnetic Resonance Image Acquisition

T1-weighted MRI volumetric brain scans were performed on 703 participants as per the GENFI protocol.^10^ 55 images were removed because they either failed the quality control check for motion and scanner artifacts or an abnormal finding was found in the form of significant vascular disease or other structural brain lesions. Subsequently, 648 scans were included in the analysis: 217 controls, 184 *C9orf72* expansion carriers, 172 *GRN* mutation carriers, and 75 *MAPT* mutation carriers.

### Statistical Analysis

#### Healthy Controls

To explore the normative performance on the tasks in the control group, percentile scores and cumulative frequencies were calculated for each, and a lower 5th percentile was generated to indicate an abnormal score. *t* Tests were performed to assess any differences on each of the tasks that were normally distributed and Mann-Whitney U tests for those that were not. Correlations between task performance and both age and education were calculated using Pearson correlation for normally distributed data and Spearman rank correlation for those that were not. Linear regressions were performed to assess the impact of language (i.e., the language spoken by the participant) on each of the tasks within the control group. Pairwise post hoc comparisons were performed to assess the difference between the groups if the overall model was significant. For data that were not normally distributed, bootstrapping with 2000 replications was used. All analyses were performed using Stata/IC (version 14.2).

#### Mutation Carriers

Multiple linear regressions were performed to assess performance on the tasks in each of the groups. Age, sex, education, and language were included in the models as covariates. Post hoc pairwise comparisons were calculated to assess the differences between the groups. For data that were not normal, bootstrapping with 2000 replications was used and the 95% bootstrapped CIs reported.

A Pearson correlational analysis was conducted on each of the tasks to measure the association with disease severity and task performance using the CDR plus NACC FTLD SB score. For data that were not normally distributed, Spearman rank correlation (rho) was used instead.

To assess the impact of phenotype in the symptomatic groups, participants were grouped into bvFTD,^18^ PPA,^13^ and FTD-ALS.^14^ Other phenotypes were not included in the analysis because of their low numbers. A linear regression was performed and included age, sex, and education as covariates in the model. For data that were not normally distributed, the model was bootstrapped (2000 replications).

#### Structural Brain Imaging Analysis

An automated atlas segmentation propagation and label fusion strategy—Geodesic Information Flow (GIF)^19^—was used on the T1-weighted volumetric MRI scans to generate brain volumes of regions of interest known to be involved in executive function^3,20^: the orbitofrontal cortex (OFC), dorsolateral prefrontal cortex (DLPFC), ventromedial prefrontal cortex, parietal lobe, and striatum. All the individual regional volumes were expressed as a percentage of total intracranial volume, as computed with SPM12 (Statistical Parametric Mapping, Welcome Trust Centre for Neuroimaging, London, United Kingdom) running under Matlab R2014b (Mathworks, USA:^21^). Using RStudio (version 1.2.1335, 2009–2019), partial correlations were performed to investigate the association between the brain regions and score on each of the executive function tasks, while taking into consideration disease severity as measured using the CDR plus NACC FTLD SB score, as well as the age of the participant.

### Data Availability

The data sets generated and/or analyzed during this study are not publicly available because the conditions of our ethical approval do not permit public archiving of individual anonymized data, but are available from the corresponding author on reasonable request.

## Results

### Healthy Controls

#### Age

Increasing age correlated with worse performance on the DSST (*r* = −0.4, *p* < 0.001); D-KEFS: Color (Rho = 0.3, *p* < 0.001); D-KEFS: Word (Rho = 0.1, *p* = 0.049); D-KEFS: Ink (Rho = 0.3, *p* < 0.001); TMT A (Rho = 0.4, *p* < 0.001); and TMT B (Rho = 0.3, *p* < 0.001) but not the DSB. Performance across each decade is summarized in eTable 2.

#### Sex

There was a significant effect of sex on the DSST (T = 3.9, *p* < 0.001, with women scoring higher) and TMT B (U = −2.1, *p* = 0.035, with women performing quicker). No other differences were found between men and women on any of the other tasks (eTable 3).

#### Education

Across all the tasks, there was a significant influence of education on task performance in the control group, with higher levels of education associated with better task performance (DSST: *r* = 0.4, *p* < 0.001; DSB: *r* = 0.3, *p* < 0.001; D-KEFS: Color: Rho = −0.2, *p* = 0.004, D-KEFS: Word: Rho = −0.2, *p* = 0.004; D-KEFS: Ink: Rho = −0.2, *p* < 0.001; TMT A: Rho = −0.1, *p* = 0.028; TMT B: Rho = −0.3, *p* < 0.001).

#### Language

Only the D-KEFS: Word and Ink tasks saw an overall influence of language on performance (D-KEFS: Word: χ^2^ (7) = 20.2, *p* = 0.005; D-KEFS: Ink: χ^2^ (7) = 15.4, *p* = 0.030, *r*^2^ = 0.056) (eTable 4).

#### Percentile Scores

Normative percentile scores were calculated on each of the tasks using the control data (eTable 5). A score less than 38 on the DSST and less than 3 on the DSB task would be considered abnormal (<5th percentile). If it took more than 40 seconds, 31 seconds, and 71 seconds to complete the D-KEFS: Color, Word, and Ink tasks, respectively, and more than 48 seconds and 125 seconds for the TMT A and B tasks, respectively, the participant's performance would also be considered abnormal (<5th percentile).

### Mutation Carriers

#### Group Comparisons

The means and SDs for the scores on the executive function tasks in each of the mutation carrier groups are listed in Table 1 and Figure. The differences between the groups are presented in eTables 6–12 and eFigure 1.

**Figure:**
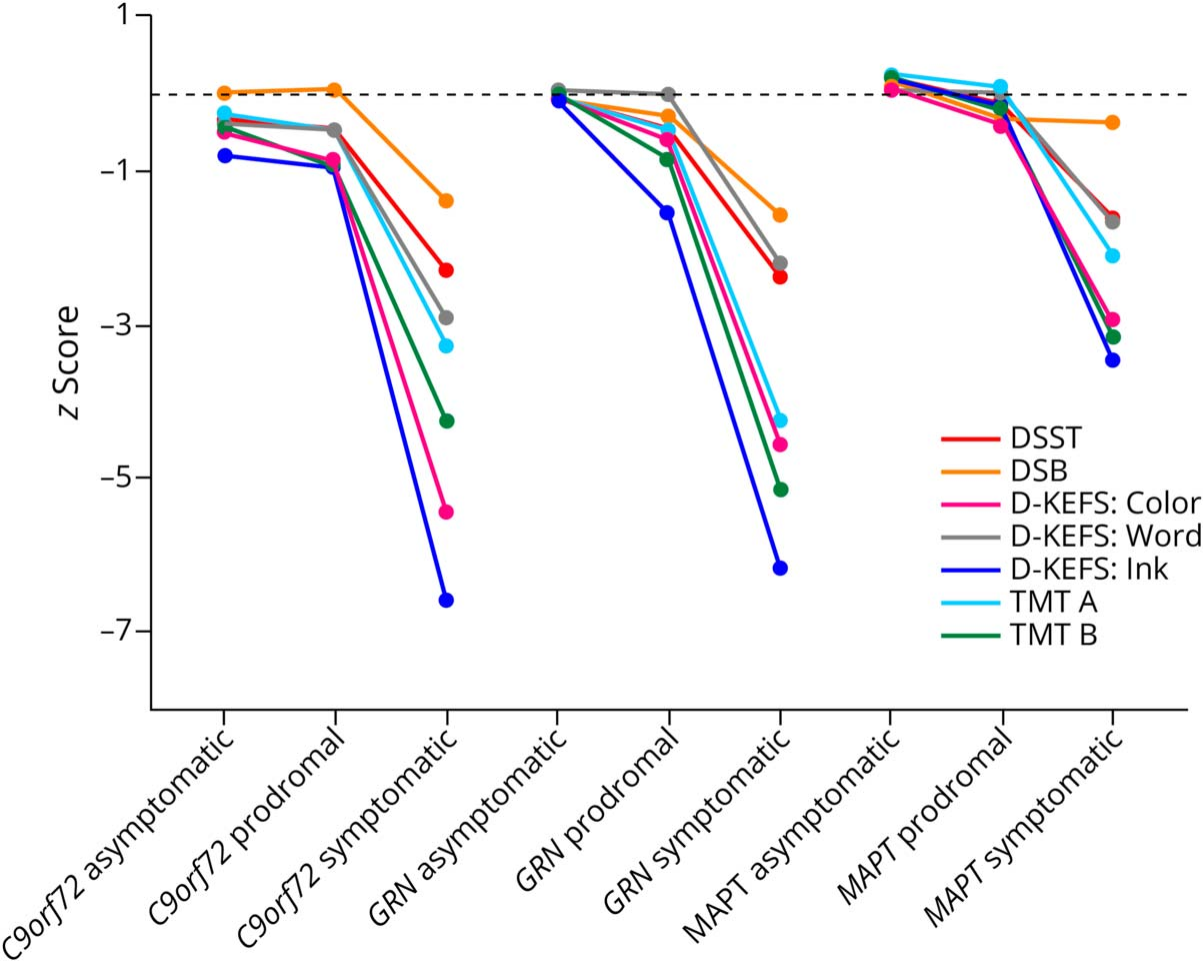
Performance of Mutation Carrier Groups on Each Executive Function Task Expressed as a Z-Score to Allow Comparison Across Tasks DSST = Wechsler Adult Intelligence Scale-Revised Digit Symbol substitution task; DSB: Weschler Memory Scale-Revised Digit Span Backwards; D-KEFS: Delis Kaplan Executive Function System (Color-Word Interference Test – Color = Color naming, Word = Word naming, Ink = Ink color naming); TMT: Trail Making Test (A = Part A; B = Part B).

All 3 symptomatic mutation carrier groups were significantly impaired on all executive function tasks compared with controls, as well as when compared with their asymptomatic and prodromal genetic groups, except for on the DSB task where no differences were seen between the different *MAPT* groups.

The *C9orf72* prodromal group was significantly impaired in comparison with the controls on the D-KEFS: Color and Ink tasks and TMT B, with a trend toward a poorer performance on the DSST (*p* = 0.066) and TMT A (*p* = 0.099). No differences were seen between the prodromal *GRN* or *MAPT* groups and controls on any of the tasks.

The *C9orf72* asymptomatic group was significantly impaired compared with controls on all tasks, except for the DSB and D-KEFS: Word task. No differences were seen between the asymptomatic *GRN* or *MAPT* groups and controls on any of the tasks.

When comparing between the genetic groups at each disease severity stage, for symptomatic mutation carriers, the *C9orf72* group performed significantly worse than the *MAPT* group on the DSB, DSST, and D-KEFS: Color and Ink tasks while the *GRN* symptomatic group performed worse than the *MAPT* symptomatic carriers on the DSB, DSST, and TMT A tasks; for prodromal mutation carriers, the *C9orf72* group performed significantly worse than the *MAPT* group on the D-KEFS: Ink task; for asymptomatic mutation carriers, the *C9orf72* group performed significantly worse than the other 2 groups on the DSST, D-KEFS: Ink, and TMT B tasks as well as worse than the *GRN* group on the D-KEFS: Color and TMT A tasks.

#### Correlation With Disease Severity

All tasks significantly correlated with disease severity as measured using the CDR plus NACC FTLD-SB score in each of the genetic groups (Table 2).

**Table 2:** Correlations of Each Task With Disease Severity as Measured Using the CDR Plus NACC FTLD-SB (for the 3 Mutation Carrier Groups)

Task	Correlation coefficient	C9orf72		GRN		MAPT	
DSST	*r*	−0.69	<0.001	−0.68	<0.001	−0.62	<0.001
DSB	*r*	−0.60	<0.001	−0.50	<0.001	−0.30	<0.001
D-KEFS: Color	Rho	0.60	<0.001	0.50	<0.001	0.50	<0.001
D-KEFS: Word	Rho	0.50	<0.001	0.30	<0.001	0.40	0.001
D-KEFS: Ink	Rho	0.60	<0.001	0.50	<0.001	0.50	0.005
TMT A	Rho	0.60	<0.001	0.50	<0.001	0.60	<0.001
TMT B	Rho	0.60	<0.001	0.50	<0.001	0.60	<0.001

CDR with NACC FTLD–SB: CDR. Dementia Staging Instrument with the National Alzheimer’s Coordinating Center Frontotemporal Lobar Degeneration component Sum-of-Boxes score; DSST: Wechsler Adult Intelligence Scale-Revised Digit Symbol substitution task; DSB: Weschler Memory Scale-Revised Digit Span Backwards; D-KEFS: Delis Kaplan Executive Function System (Color-Word Interference Test—Color = Color naming, Word = Word naming, Ink = Ink color naming); TMT: Trail Making Test (A = Part A; B = Part B). The DSST and DSB were normally distributed and so Pearson correlations were performed. For the remainder, Spearman rank correlations were performed.

#### Phenotypic Analysis

When compared with controls, all phenotypic groups were significantly impaired on all tasks of executive function (all *p* < 0.001, except for the FTD-ALS group on the TMT A where *p* = 0.016). The PPA group scored significantly worse on the DSB task than the bvFTD group (adjusted mean difference [AMD] = −1.5, *p* = 0.011) and FTD-ALS group (AMD = −1.5, *p* = 0.012) and significantly worse than the bvFTD group on the TMT B (bvFTD: AMD = 56.2, *p* = 0.011).

#### Imaging Analysis

The partial correlations between task score and regional brain volume for each group are presented in eTable 13. For *C9orf72* expansion carriers, correlations between task performance and regional brain volumes were seen with the DLPFC (DSST, DSB, and D-KEFS: Ink) and parietal cortex (DSST and TMT B) and with the striatum on the DSST. For *GRN* mutation carriers, fewer significant correlations were seen: right OFC with Trail Making Test Part B and DLPFC with D-KEFS: Color and Ink tasks. For *MAPT* mutation carriers, significant correlations were seen mainly with the DLPFC (D-KEFS: Color and Ink and TMT B) and striatum (DSST and D-KEFS: Color), as well as the left orbitfrontal cortex with D-KEFS: Ink.

## Discussion

In this study, the executive function abilities of a large cohort of individuals with genetic FTD were comprehensively assessed. It demonstrates that executive dysfunction can be present in both individuals who are symptomatic of familial FTD across all 3 genetic groups, as well as in those with a *C9orf72* expansion who are asymptomatic and prodromal. Neither the *GRN* nor the *MAPT* asymptomatic or prodromal mutation carriers showed significant differences on the executive function tasks compared with the control group suggesting that executive function changes occur later in the disorder than in *C9orf72*-associated FTD.

The control group data indicate that of all the demographic covariates, age and education were most associated with executive function score. This was to be expected because many studies have found that older age and lower education leads to greater impairment on many of the executive function tasks.^22-26^ Only 2 tasks showed an effect of sex on score, which is supported by previous work, with women performing better on the DSST, while men achieved better scores on the TMT B task.^27-29^ Finally, language did also have an influence on task performance, affecting those tests that had an element of language to them: the D-KEFS: Word and Ink tasks. This may well be due to the slight variation in the length of words in the different languages used in the study, which may take longer to pronounce. Further to note is the differences in sample sizes across the control groups, which may also have influenced the findings in this study.

As expected, and in line with the previous literature, the symptomatic individuals displayed executive dysfunction irrespective of genetic group.^4-9^ This was the case when they were compared with both the control group and their asymptomatic and prodromal counterparts. The only exception to this was on the DSB task where the symptomatic *MAPT* mutation carriers were not significantly impaired.

The asymptomatic and prodromal *C9orf72* groups displayed early executive dysfunction in tasks assessing inhibition, set switching/cognitive flexibility, processing speed and general cognitive function, while working memory abilities were less affected early on. This is in line with a recent study that produced a cognitive composite for each of the genetic mutations that displayed widespread cognitive dysfunction in *C9orf72* mutation carriers, including deficits in executive function.^30^ Although 2 of the tasks in the prodromal group did not show a significant difference compared with controls despite this being seen in the asymptomatic group, it is likely that this was due to the smaller sample size in the prodromal group and thus was not sufficiently powered to detect a deficit. Differences at these early stages are small and are likely to represent changes in some individuals but not others; future work will focus on investigating this further.

Neither the *GRN* nor *MAPT* asymptomatic or prodromal carriers displayed any early executive dysfunction when compared with the controls. For the *GRN* mutation carriers, this is consistent with previous work that has demonstrated a rapid decline in symptoms in *GRN* mutation carriers during the first year of diagnosis compared with *C9orf72* and *MAPT* mutation carriers,^8^ with a rapid increase in atrophy rates after symptom onset,^31^ and the highest NfL levels of the 3 genetic groups in the symptomatic period, despite showing no difference to controls in the asymptomatic or prodromal period.^32^ So, despite the clear deficit in executive function in the symptomatic phase of the disease, this work suggests that it is a problem presenting later in the disease course and thus may not be a useful marker of disease progression in primary or secondary prevention clinical trials. By contrast, it is likely that executive function is less affected in *MAPT* mutation carriers. Overall, the performance on the executive function tasks by the *MAPT* symptomatic mutation carriers was better than those of the other symptomatic genetic groups, with no significant difference being seen between the *MAPT* symptomatic group and the controls on the DSB task. The atrophy pattern in *MAPT* mutation carriers is much more localized with significant atrophy in the hippocampus, amygdala, and temporal lobes.^20,33^ These are the regions usually associated with language and memory abilities, of which impairments are present early on in *MAPT* mutation carriers.^30,34,35^

It was expected that individuals with a bvFTD diagnosis would perform worse than all other phenotypes on these executive tasks because it is a key diagnostic feature^12^ and there has been much evidence to support this in the sporadic literature.^36-44^ However, we find here that this was not the case on all tasks of executive function. Performance on the TMT B task was worst in those with a PPA diagnosis compared with those individuals with bvFTD, and performance on the DSB task was also worse in those with PPA compared with those with bvFTD or an ALS diagnosis. It is possible that this is because the DSB and TMT B tasks require some aspect of language function to be able to complete the test and thus are not solely executive function tasks. The DSB task requires participants to access their lexicon to generate and produce number words, which is a particular problem if the individual is nonfluent. Furthermore, the TMT B task requires knowledge of the alphabet. The human visual word form area or letterbox is found within the temporal lobe,^45^ a key region involved in PPA.^46^ It is therefore likely that executive function is not necessarily more impaired in PPA than it is in bvFTD and ALS, but rather the tasks are not solely executive tasks and so they are performing poorly in them due to the underlying language requirements. Despite this, all 3 phenotypes (bvFTD, PPA, and ALS) displayed executive dysfunction when compared with controls, which was expected and in line with prior work.^38,47,48^

When looking at the mutation carriers as a whole, a clear decline in executive function is seen on all tasks for each genetic group as the disease progresses when disease severity is measured using the CDR plus the NACC-FTLD-SB score. This is consistent with previous work that demonstrates that function declines with disease severity in FTD.^49,50^

The region-of-interest analysis revealed that in the *C9orf72* mutation carriers, executive function score was associated with atrophy in the DLPFC as well as the parietal lobe mainly. This is consistent with previous literature showing key involvement of the DLPFC in executive function abilities,^51-53^ a region that is part of a wider frontoparietal executive function network.^54-57^ The DSST was associated with atrophy in multiple regions, likely because it assesses multiple cognitive processes including processing speed, working memory, and reasoning.^58^ In contrast to the *C9orf72* neural correlates, there were fewer correlates with the *GRN* mutation groups but consistent with involvement of the frontal lobe, there were some associations with both the orbitofrontal and dorsolateral prefrontal cortices. Finally, when looking at the neural correlates of the *MAPT* mutation carriers, similar to the *GRN* mutation carriers, there is involvement of some frontal regions on a few of the tasks: OFC on the D-KEFS: Ink task and the DLPFC on the TMT B and the D-KEFS: Color and Ink tasks. Striatal atrophy was also correlated with scores on the DSST and D-KEFS: Color tasks, a region highly connected to the frontal lobe and well known to be associated with executive dysfunction when impaired.^59,60^

Limitations to the study include the relatively small number of individuals investigated when breaking down the cohort into smaller groups. Further work is required to increase the sample size ensuring greater power and confidence in the results. A second limitation is the paucity of language-specific tasks available in the GENFI cognitive battery—future studies would examine the association of executive and language function across the different phenotypes, allowing us to break down the PPA group into individual subgroups and understand further how language may be affecting performance, especially in the *GRN* mutation carriers. Finally, we are currently not able to distinguish whether executive function deficits seen early in the disease process (particularly in the *C9orf72* group) are acquired as part of a prodromal neurodegenerative process or present as part of a neurodevelopmental disorder—future longitudinal studies and investigation of children with such variants will be required to understand this.

To conclude, this study comprehensively assessed executive function abilities in a large cohort of individuals with genetic forms of FTD. It is clear that some individuals with *C9orf72* expansions have difficulties with executive function from a very early stage in the disease and this continues to deteriorate with disease severity. By contrast, executive dysfunction occurs in the later stages of the disease in *GRN* and *MAPT* mutation carriers. While it is assumed that executive dysfunction is a core feature of FTD, it appears that not all tasks measuring executive function do so equally across the genetic groups and so great care and consideration should be given when thinking about what tasks should be included as outcome measures in upcoming clinical trials based on the target genetic group and stage of the individuals being recruited.

## Glossary

AMD: adjusted mean difference
CDR: Clinical Dementia Rating
DLPFC: dorsolateral prefrontal cortex
DSB: Digit Span Backwards
DSST: Digit Symbol substitution task
FTD: frontotemporal dementia
GENFI: Genetic FTD Initiative
GIF: Geodesic Information Flow
GRN: progranulin
MAPT: microtubule-associated protein tau
OFC: orbitofrontal cortex
SB: sum of boxes
TMT: Trail Making Test
WMS-R: Weschler Memory Scale-Revised
